# Sensitivity, Resistance Risk Assessment of *Bipolaris oryzae* to Three DMI Fungicides and Their Control Efficacy Against Banana Eyespot Disease in Hainan Island, China

**DOI:** 10.3390/jof12080546

**Published:** 2026-07-23

**Authors:** Yanxiang Qi, Zhaojing Zhang, Hong Zhao, Xuelian Xu, Xin Zhang

**Affiliations:** 1 Hainan Provincial Key Laboratory of Pests Detection and Control for Tropical Agriculture, Environment and Plant Protection Institute, Chinese Academy of Tropical Agricultural Sciences, Haikou 571101, China; xuelian_x@163.com; 2 Jinan Lefeng Crop Science Co., Ltd., Jinan 251600, China; 15092851239a@sina.com; 3 Nanjing Shuxi Intelligent Technology Co., Ltd., Nanjing 210012, China; zhaohong0430@163.com

**Keywords:** banana eyespot, *Bipolaris oryzae*, demethylation inhibitor fungicide, sensitivity, resistance risk, control efficacy

## Abstract

*Bipolaris oryzae* was recently identified as a new species causing eyespot disease of banana in China. Propiconazole, flusilazole and tebuconazole, belonging to demethylation inhibitor (DMI) fungicides, are commonly used for controlling this disease in China. In this study, the propiconazole, flusilazole and tebuconazole sensitivity of 108 *B. oryzae* isolates collected from banana leaf samples in different locations of Hainan Island, China, in 2021–2023 was determined by mycelial growth inhibition, and the control efficacy of the three DMI fungicides against *B. oryzae* was assayed in pot experiments. The sensitivity frequency distributions of 108 *B. oryzae* isolates to propiconazole, flusilazole and tebuconazole indicated the emergence of subpopulations with reduced sensitivity, with EC_50_ values ranging from 0.029 to 3.495 μg/mL, 0.008 to 3.665 μg/mL, and 0.022 to 8.288 μg/mL, with means and standard errors of 0.826 ± 0.059 μg/mL, 0.204 ± 0.036 μg/mL and 0.952 ± 0.113 μg/mL, respectively. However, the sensitivity distributions of 82.41%, 82.41%, and 80.56% of *B. oryzae* isolates at the main peak, respectively, to the three fungicides fitted to a normal distribution. Thus, the mean EC_50_ values of 0.594 ± 0.032 μg/mL, 0.118 ± 0.007 μg/mL and 0.574 ± 0.033 μg/mL of these isolates could be used as the baseline for testing the sensitivity of *B. oryzae* to propiconazole, flusilazole and tebuconazole, respectively. No significant sensitivity differences to each fungicide among different geographical populations were observed, but a statistically significant and positive association in sensitivity between all three fungicides existed. Seven unstably inherited DMI-resistant isolates all exhibited lower fitness levels than those of the sensitive isolates, suggesting a low risk of resistance to the three DMI fungicides in *B. oryzae.* Three fungicides applied separately exhibited 100% protective control efficacy against *B. oryzae* and less than 40% curative control efficacy in pot experiments. Therefore, propiconazole, flusilazole and tebuconazole should be used as protective rather than curative fungicides. These results provide essential information on the evolution of DMI resistance in *B. oryzae* in Hainan Island, China, and may serve as a reference point in the management of *B. oryzae* and future DMI resistance monitoring programs.

## 1. Introduction

The ascomycete genus *Bipolaris* contains a wide variety of important plant pathogens with a worldwide distribution, which can produce leaf spots, leaf blights, melting outs, root rots, and foot rots on an array of plant hosts, including banana and economically important hosts worldwide [1,2]. *Bipolaris gigantea* (syn. *Drechslera gigantea*) and *Bipolaris sacchari* are known pathogens of banana eyespot in Jamaica, Uganda and Brazil [3,4,5,6]. In 2021 and 2022, another *Bipolaris* species was first isolated from leaves of the banana cultivar ‘Huangdi banana’ (*Musa acuminata* AA Sucrier cv. Pisang Mas) with eyespot symptoms in Hainan Island, China, and these isolates were identified as *Bipolaris oryzae* based on morphological and molecular analyses as well as a pathogenicity test [2].

Banana (*Musa* sp.), which originated in South-East Asia, is an important staple and economic crop in tropical and subtropical regions of the world, and China is the second-largest producer and consumer of banana in the world [7]. ‘Huangdi banana’, also known as ‘Gonjiao’ or ‘Gold banana’, is a cherished banana cultivar well known for its small and sweet fruit, bright color, and unique taste; its average price is CNY 10–14 per kg, above that for a typical banana, CNY 4–6 per kg. The planting area in Hainan Island has reached 4.07 × 10^3^ hectares, accounting for 81.33% of the total planting area in China [8], among which Chengmai is known as the home of the ‘Huangdi banana’ in China due to having the largest planting area. ‘Huangdi banana’, as a Fusarium wilt-resistant banana cultivar, shows susceptibility to foliage diseases caused by various fungal pathogens [9]. Based on our investigation, the rates of diseased plants and leaves caused by *B. oryzae* in some ‘Huangdi banana’ plantations can reach 100% and 85%, respectively, during the rainy season and humid weather, with estimated yield losses of 10–15% [8]. However, other banana cultivars, such as ‘Brazil’, ‘Formosana’ and ‘Nantianhuang’ (*M. acuminata* AAA Cavendish), ‘Guangfen 1’ (*Musa* spp. ABB Pisang Awak), and ‘Dongguan dajiao’ (*Musa* spp. ABB Dajiao), were lightly affected, or even not affected by *B. oryzae*. Banana is a large, perennial herb, and continuous cropping without a fallow period will result in the build-up of plant pathogens. Eyespot, as a new disease of banana in Hainan Island, China, has the potential to be a major yield-limiting disease in areas producing ‘Huangdi banana’ due to its ever-increasing disease incidence.

Management strategies to control foliage diseases of banana rely heavily on fungicide applications, due to the lack of appropriate commercial resistant varieties, among which sterol demethylation inhibitor (DMI) fungicides with a site-specific mode of action are the most widely used for controlling foliage diseases of banana worldwide [10]. DMI fungicides inhibit fungal growth by interfering with ergosterol biosynthesis through inhibition of the sterol 14 alpha-demethylase cytochrome P450 (*cyp51*) [11,12]. Resistance to DMI fungicides in some plant pathogens has been reported to be of a quantitative nature characterized by slow shifts in sensitivity toward resistance, which is commonly associated with overexpression of the *cyp51* gene encoding the target protein of DMI fungicides [13,14,15,16,17]. Despite their effectiveness in controlling foliage diseases of banana, the extensive use of DMI fungicides, including repeated applications within the same growing season, has increased the risk of fungal populations developing reduced sensitivity or formation resistance [17,18,19,20]. Reduced sensitivity or formation resistance to three DMI fungicides (propiconazole, flusilazole or tebuconazole) has been reported in pathogens of banana Sigatoka leaf spot complex, such as *Pseudocercospora fijiensis* (black Sigatoka pathogen) and *Pseudocercospora muase* (yellow Sigatoka pathogen) [10,17,19].

DMI fungicides such as propiconazole, flusilazole and tebuconazole have been registered in China to control various plant diseases, including foliage diseases of banana (China Pesticide Information Network, http://www.chinapesticide.org.cn (accessed on 16 January 2024)). Despite the increasing importance of fungicides in managing eyespot on banana, data on the sensitivity of *B. oryzae*, causing banana eyespot, to propiconazole, flusilazole and tebuconazole are still unclear, and no case of resistance to the fungicides has been reported in China. In addition, few studies have reported their activity and efficacy against *B. oryzae*.

The objectives of this study were to provide references to studies on the efficacy of fungicides against *B. oryzae* by (a) evaluating the sensitivity of field isolates of *B. oryzae* to three DMI fungicides (propiconazole, flusilazole and tebuconazole) and establishing the sensitivity baselines; (b) clarifying the sensitivity correlation among propiconazole, flusilazole and tebuconazole; (c) assessing the risk of occurrence of resistance to the three DMI fungicides in *B. oryzae* by evaluating the resistant stability in *B. oryzae*, and comparing the fitness level of the sensitive and the DMI-resistant isolates; and (d) testing the protective and curative activities of propiconazole, flusilazole and tebuconazole against eyespot disease on banana in pot experiments.

## 2. Materials and Methods

### 2.1. Collection of B. oryzae Isolates

All isolates of *B. oryzae* used in this study were isolated from 32 banana plantations planted with the banana cultivar ‘Huangdi banana’ (*Musa acuminata* AA Sucrier cv. Pisang Mas) in Hainan Island, China, in 2021–2023. The orchards are at least 10 km apart, with many years of history of propiconazole, flusilazole and tebuconazole exposure. In each plantation, three to five banana leaves (100 mm^2^) with eyespot symptoms were randomly collected, sealed in a sterile plastic bag and taken back to the laboratory, and then stored at 4 °C. All laboratory experiments were conducted in a laminar flow cabin. To isolate *B. oryzae,* symptomatic leaves (15 mm^2^) were surface disinfected in 70% ethanol (10 s), rinsed in sterile water three times, and transferred to potato dextrose agar (PDA, 200 g/L potato, 20 g/L dextrose, and 18 g/L agar) plates amended with streptomycin sulfate at 28 °C for 3 days. Pure cultures were obtained by transfer of a single hyphal tip to the fresh PDA plate, and incubated at 28 °C for 3 days. Isolates were identified to be *B. oryzae* as described by Zhao et al. [2]. A total of 108 isolates were obtained and preserved on PDA slants at 4 °C for 2 to 4 weeks and in 20% glycerol at −80 °C for long-term storage.

### 2.2. Fungicides

Technical-grade fungicides included propiconazole (95% active ingredient [a.i.]; Hubei Huiheyuan Chemical Co., Ltd., Wuhan, China), flusilazole (95% a.i.; Hubei Huiheyuan Chemical Co., Ltd., Wuhan, China), and tebuconazole (97% a.i.; Hubei Huiheyuan Chemical Co., Ltd., Wuhan, China). Formulated fungicides included propiconazole emulsifiable concentrate (EC) (250 g/L; Shandong Weifang Shuangxing Pesticides Co., Ltd., Weifang, China), flusilazole EC (400 g/L; Xingnong Pharmaceutical (China) Co., Ltd., Shanghai, China) and tebuconazole wettable powder (WP) (25% Yancheng Shuangning Agrochemical Co., Ltd., Yancheng, China).

### 2.3. Sensitivity of B. oryzae to Three DMI Fungicides In Vitro

To determine the sensitivity to propiconazole, flusilazole and tebuconazole, the 108 *B. oryzae* isolates were used to determine the EC_50_ values (the concentration of the fungicide causing a 50% reduction in the growth compared with an unamended control) using mycelial growth assay. Stock solutions of three technical-grade fungicides were prepared by dissolving each fungicide in dimethyl sulfoxide (DMSO) at a concentration of 2 × 10^4^ μg/mL, and preserved at 4 °C. Serial dilutions were prepared using sterile double-distilled water (ddH_2_O). PDA plates containing propiconazole or tebuconazole were prepared by mixing 90 mL medium (at ~50 °C) with 10 mL propiconazole or tebuconazole dilution of various concentrations to obtain final concentrations of 0, 0.1, 0.5, 1.0, 5.0, and 10.0 μg/mL, and the final DMSO concentration was adjusted to 0.1% (vol/vol). PDA plates containing flusilazole were prepared as described above to obtain final concentrations of 0, 0.025, 0.05, 0.1, 0.5, and 1.0 μg/mL. The fungicide-free PDA plates supplemented with 0.1% (vol/vol) DMSO served as controls.

An inverted 8 mm diameter mycelial plug, cut from the actively growing margin of a 3-day-old colony, was transferred to the center of 9 cm Petri dishes containing fungicide with different concentrations. PDA plates were incubated at 28 °C in the dark for 3 days, and the mean diameter (minus the diameter of the inoculation plug) was measured in two perpendicular directions for each treatment and expressed as percentage growth inhibition. Each treatment consisted of three replicates, and the test was repeated twice. The percent inhibition of mycelial growth (PIMG) was calculated by the formula

PIMG = [(C − T)/C] × 100%,

where C and T refer to mean colony diameter of the control and mean colony diameter of the treatment, respectively.

EC_50_ values, correlation coefficients (r), and virulence regression equations were calculated for each isolate by performing the linear regression analysis of probit values of percentage growth inhibitions against logarithms of fungicide concentrations. The variation factor (VF) was calculated as the ratio of the maximum EC_50_ value (Maximum EC_50_) relative to that of the minimum (Minimum EC_50_). The sensitivity baseline was determined by calculating sensitivity distributions (frequency distributions of EC_50_ values).

The EC_50_ values were used to estimate the resistance factor (RF), which indicates the sensitivity level of the tested isolates. The RF was calculated by the ratio of the EC_50_ values of resistant isolates relative to the mean EC_50_ values of sensitive isolates. RF (zero-to 5-fold) indicates no resistance (sensitive, S), RF (5- to 10-fold) indicates low resistance (LR), and RF (10- to 40-fold) indicates moderate resistance (MR) [21]. The resistance frequency was calculated via the following formula:

resistance frequency  = number of fungicide-resistant isolates/total number of isolates × 100%.

### 2.4. Correlation Analysis of B. oryzae Sensitivity to Three DMI Fungicides

All 108 isolates tested were chosen and the EC_50_ values of these isolates to propiconazole, flusilazole and tebuconazole were converted to the logarithm (lgEC_50_). The cross-resistance relationship among propiconazole, flusilazole and tebuconazole was analyzed by correlation analysis [22].

### 2.5. Stability of Field-Resistant Isolates of B. oryzae

From the results of the propiconazole, flusilazole and tebuconazole sensitivity tests, all seven field-resistant isolates of *B. oryzae*, including one propiconazole-resistant isolate, two flusilazole-resistant isolates, three tebuconazole-resistant isolates, and one isolate resistant to both flusilazole and tebuconazole, were selected for stability evaluation. The stability test was performed on fungicide-free PDA. An inverted mycelial plug (diameter of 8 mm) from the edge of a 3-day-old resistant isolate of *B. oryzae* was transferred to the center of 9 cm Petri dishes containing the fresh fungicide-free PDA, and the plates were incubated at 28 °C for 3 days in the dark. After culturing the seven resistant isolates on fungicide-free PDA plates for 8 consecutive generations, EC_50_ values for three technical-grade fungicides were determined after the last transfer. The determination method and fungicide concentrations remained constant over the duration of the experiment as described above. The EC_50_ values were compared with the initial EC_50_ values to the evaluate the stability of the fungicide sensitivity. Each treatment consisted of three replicates, and the experiment was repeated twice.

### 2.6. Fitness Evaluation of Field-Resistant Isolates of B. oryzae

Seven field-resistant and two sensitivity isolates (sensitive to all three fungicides) were evaluated in terms of mycelial growth rate, mycelial dry weight, and pathogenicity. The mycelial growth rate test was conducted on fungicide-free PDA. An inverted mycelial plug (diameter of 8 mm), cut from the margin of a 3-day-old colony, was transferred to the center of Petri dishes (diameter of 9 cm) containing the fresh fungicide-free PDA. After incubation for 3 days at 28 °C in the dark, the mean diameter (minus the diameter of the inoculation plug) was measured in two perpendicular directions for each treatment. Each treatment consisted of three replicates, and the test was repeated twice.

Mycelial dry weight test was conducted in fungicide-free potato dextrose broth (PDB, 200 g/L potato and 20 g/L dextrose) medium. Three fresh mycelial plugs (diameter of 8 mm) were transferred into a flask containing 100 mL PDB. After the flasks were shaken at 200 rpm and 28 °C in the dark for 3 days, the mycelia were filtered and washed three times with distilled water, then dried to a constant weight at 60 °C in an air drier, and the weight was recorded. Each treatment consisted of three replicates, and the experiment was repeated twice.

A pathogenicity test was conducted using plug inoculation on healthy potted 7-leaf -old ‘Huangdi banana’ plants. Two healthy leaves of the same developmental stages of each plant were selected, washed with tap water, and then surface-disinfected in a 1% sodium hypochlorite solution for 30 s, rinsed in sterile water three times, and then allowed to air-dry. Mycelial plugs with diameter of 8 mm cut from the 3-day-old *B. oryzae* colonies were placed upside down on the adaxial surface of the leaves and covered with sterile, moist cotton. For controls, leaves were inoculated with sterile PDA plugs. The inoculated leaves were wrapped individually with plastic bags to maintain high relative humidity, and the potted plants were kept at 26 °C in a greenhouse. The cotton and mycelial plugs were removed at 24 h post-inoculation, and the leaves were kept wrapped in plastic bags. After 5 days of inoculation, the diameter of each disease lesion was measured in two perpendicular directions and then averaged. The experiment was repeated twice, with six inoculation sites per leaf, two inoculated leaves on each plant, and three plants per treatment.

### 2.7. Control Efficacy of Three DMI Fungicides

The control efficacy (protective and curative) of propiconazole, flusilazole and tebuconazole against seven field-resistant and two sensitivity isolates of *B. oryzae* were evaluated on potted ‘Huangdi banana’ plants as described previously [8] with some modifications. Banana plant selection and leaf pretreatment were conducted with a similar procedure as the pathogenicity assessment above. The highest recommended doses in the field of the three formulated fungicides were used as the treatment concentrations: 500 μg/mL for 250 g/L of propiconazole EC, 66.67 μg/mL for 400 g/L of flusilazole EC, and 25 μg/mL for 25% tebuconazole WP. Stock solutions of three formulated fungicides were diluted with sterile water. Both sides of the leaves of the selected potted plants were sprayed with dilutions of propiconazole at 500 μg/mL, flusilazole at 66.67 μg/mL and tebuconazole at 25 μg/mL using a handheld sprayer until run-off. Banana plants sprayed with sterile water served as controls.

For evaluation of protective activity, treated banana leaves were air-dried for 24 h and inoculated on the adaxial surface with inverted mycelial plugs (diameter of 8 mm) from 3-day-old *B. oryzae* colonies and covered with sterile, moist cotton. For evaluation of curative activity, banana leaves were inoculated with mycelial plugs and incubated at 26 °C for 24 h, then were sprayed with formulated fungicide dilutions as described above. Subsequent processing of the inoculated leaves and potted plants was conducted to assess disease development using a similar procedure as the pathogenicity assessment above.

The disease severity (DS) was then determined by measuring the lesion diameter in two perpendicular directions 5 days after inoculation. The experiment was repeated twice, with six inoculation sites per leaf, two inoculated leaves on each plant, and three plants per treatment. The control efficacy (CE) was calculated by the formula [8].

CE = [(mean lesion diameter of the control − mean lesion diameter with the treatment)/mean lesion diameter of the control] × 100%.

### 2.8. Data Analysis

All the data were recorded using Excel software, version 2021, and analyzed using SPSS Statistics software, version 27.0 (IBM Corp., Armonk, NY, USA). The frequency distribution of EC_50_ values was assessed for normality based on the one-sample Kolmogorov–Smirnov (K-S) test. A one-way analysis of variance (ANOVA) and Duncan’s test (*p* = 0.05) were used to analyze the differences between the variables, including the EC_50_ values, fitness parameters and control efficacy. EC_50_ values were transformed to relative logarithm values, and Spearman rank correlation analysis was conducted.

## 3. Results

### 3.1. Baseline Sensitivity of B. oryzae to Three DMI Fungicides

**Figure 1:**
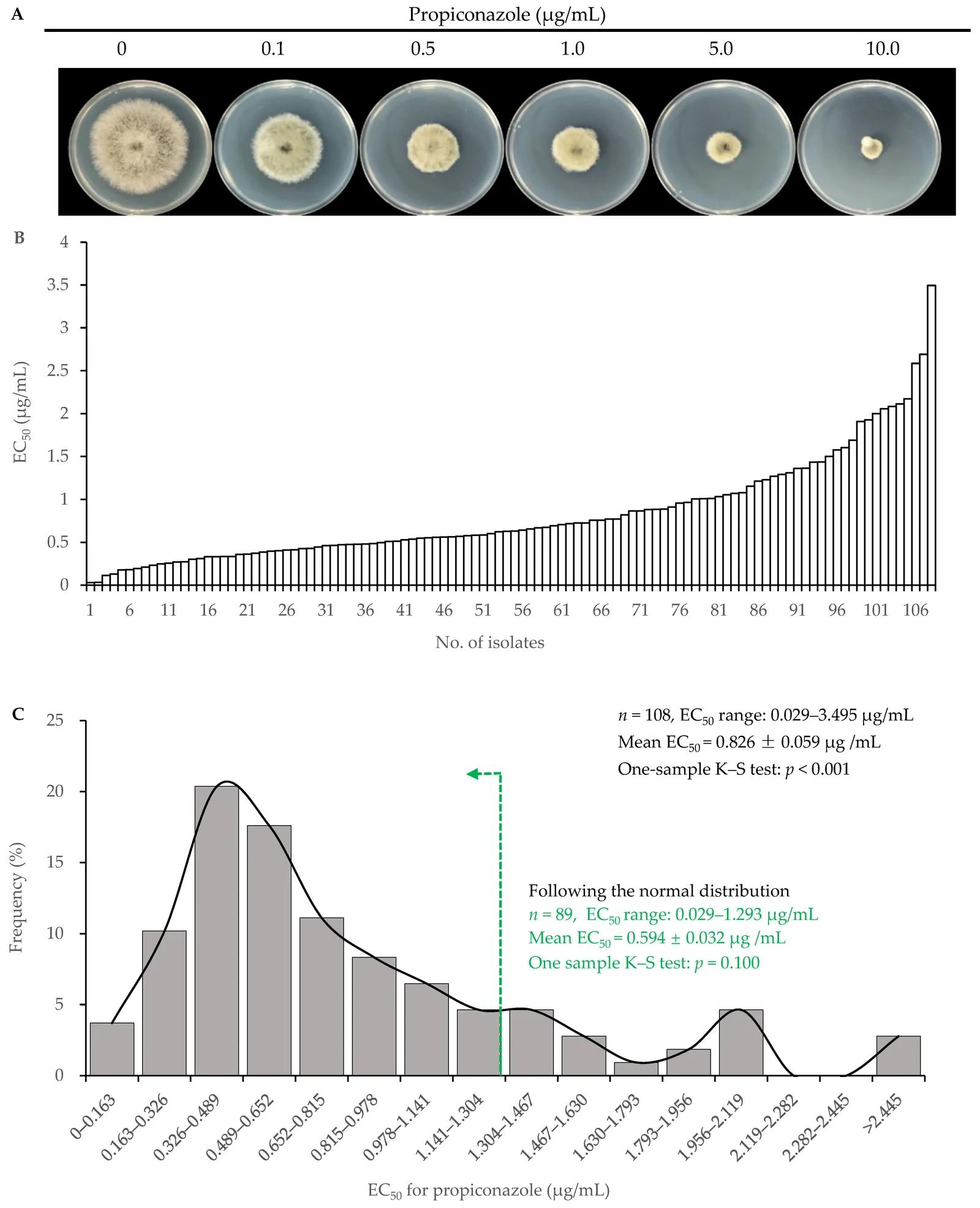
Sensitivity of *B. oryzae* to propiconazole. (**A**) Sensitivity was determined on PDA plates with 0, 0.1, 0.5, 1.0, 5.0, and 10.0 μg/mL propiconazole. (**B**) Sensitivity of 108 *B. oryzae* isolates to propiconazole. (**C**) Frequency distribution of EC_50_ values of 108 *B. oryzae* isolates to propiconazole. A one-sample Kolmogorov–Smirnov test was performed by SPSS 27.0. *p* < 0.05 means it is not normally distributed, and *p* > 0.05 means it is normally distributed.

**Figure 2:**
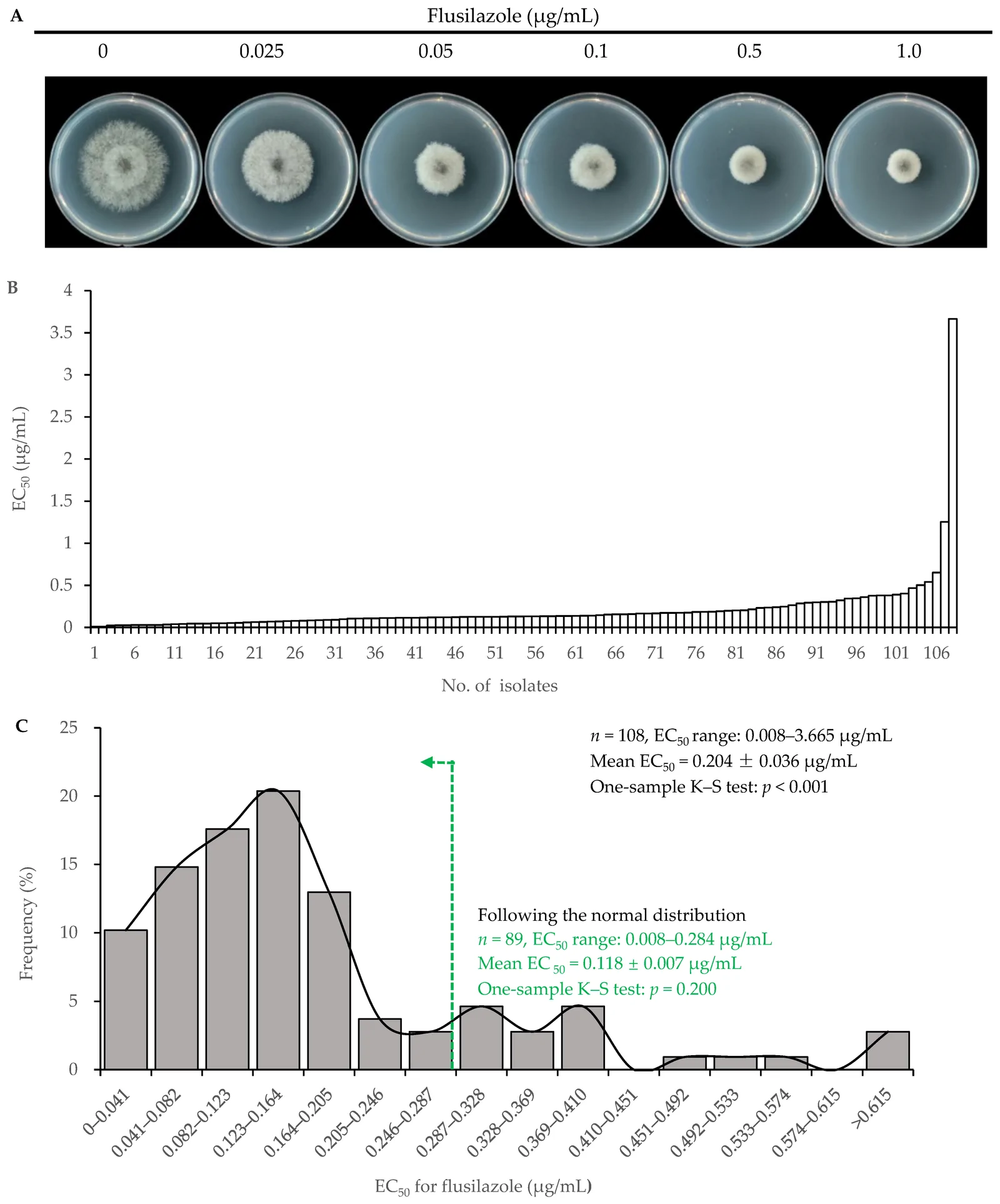
Sensitivity of *B. oryzae* to flusilazole. (**A**) Sensitivity was determined on PDA plates with 0, 0.025, 0.05, 0.1, 0.5, and 1.0 μg/mL flusilazole. (**B**) Sensitivity of 108 *B. oryzae* isolates to flusilazole. (**C**) Frequency distribution of EC_50_ values of 108 *B. oryzae* isolates to flusilazole. A one-sample Kolmogorov–Smirnov test was performed by SPSS 27.0. *p* < 0.05 means it is not normally distributed, and *p* > 0.05 means it is normally distributed.

**Figure 3:**
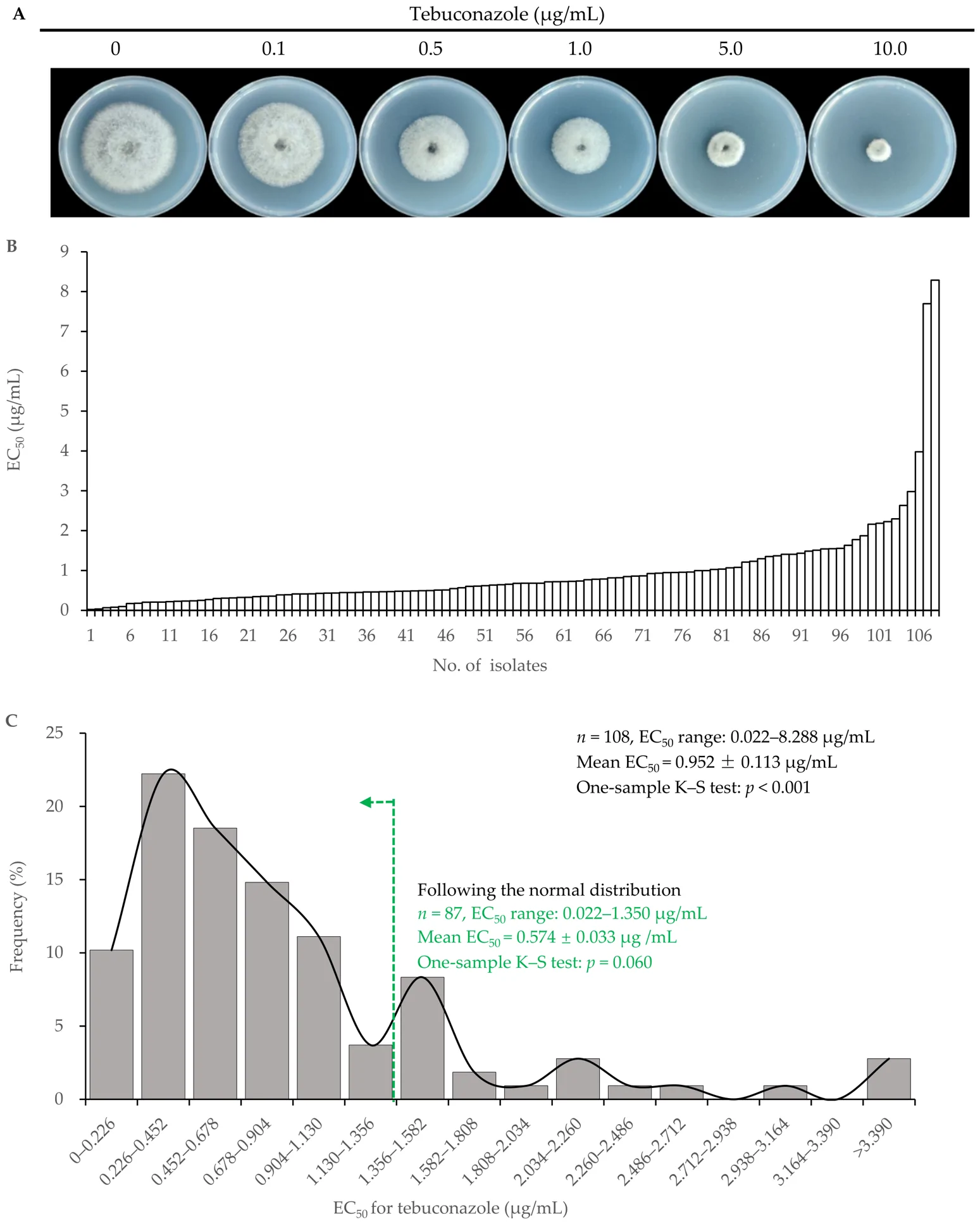
Sensitivity of *B. oryzae* to tebuconazole. (**A**) Sensitivity was determined on PDA plates with 0, 0.1, 0.5, 1.0, 5.0, and 10.0 μg/mL tebuconazole. (**B**) Sensitivity of 108 *B. oryzae* isolates to tebuconazole. (**C**) Frequency distribution of EC_50_ values of 108 *B. oryzae* isolates to tebuconazole. A one-sample Kolmogorov–Smirnov test was performed by SPSS 27.0. *p* < 0.05 means it is not normally distributed, and *p* > 0.05 means it is normally distributed.

The fungicide sensitivity of the 108 *B. oryzae* isolates from Hainan Island, China, was determined using the mycelial growth inhibition method (Figure 1A, Figure 2A and Figure 3A). EC_50_ values for the *B. oryzae* population (*n* = 108) with respect to propiconazole, flusilazole and tebuconazole ranged from 0.029 to 3.495 μg/mL, 0.008 to 3.665 μg/mL and 0.022 to 8.288 μg/mL, with means and standard errors of 0.826 ± 0.059 μg/mL, 0.204 ± 0.036 μg/mL and 0.952 ± 0.113 μg/mL, among which the VFs were 119.27-fold, 458.10-fold and 376.73-fold, respectively (Table 1). These results showed significant differences in sensitivity among individuals within the tested *B. oryzae* population (Figure 1B, Figure 2B and Figure 3B). With 0.163, 0.041 and 0.226 μg/mL as the group intervals, respectively, the EC_50_ values of the 108 *B. oryzae* isolates to propiconazole, flusilazole and tebuconazole were divided into 16 intervals. The sensitivity frequency of the EC_50_ values for propiconazole, flusilazole and tebuconazole was not normally distributed according to the one-sample Kolmogorov–Smirnov test of SPSS 27.0 (*p* < 0.001) (Figure 1C, Figure 2C and Figure 3C). There was a differentiated sensitivity of *B. oryzae* to the fungicides, and a few subpopulations with reduced sensitivity emerged. Further analysis showed the EC_50_ values of 82.41% (*n* = 89), 82.41% (*n* = 89) and 80.56% (*n* = 87) of the isolates were concentrated in the corresponding main peaks (Figure 1C, Figure 2C and Figure 3C), and the sensitivity frequency distributions were continuous unimodal curves of approximately normal distributions (*p* = 0.100, 0.200 and 0.060, respectively). Therefore, the means and standard errors of 0.594 ± 0.032 μg/mL, 0.118 ± 0.007 μg/mL and 0.574 ± 0.033 μg/mL of these isolates could be utilized as the sensitivity baseline for *B. oryzae* to propiconazole, flusilazole and tebuconazole, respectively.

### 3.2. Sensitivity Difference of B. oryzae Between Geographic Populations

Sensitivity differences of *B. oryzae* isolates to propiconazole, flusilazole and tebuconazole among geographic populations were assayed by SPSS 27.0 (Table 1). The results showed that the mean EC_50_ values for isolates of *B. oryzae* from different locations of Hainan Island were not significantly different (*F* = 1.67, *p* = 0.178; *F* = 0.07, *p* = 0.978; and *F* = 0.86, *p* = 0.463 for propiconazole, flusilazole and tebuconazole, respectively) (Table 1). There is only one isolate from Ding’an, so it was not included in the differential analysis.

### 3.3. Correlation of Sensitivity to Three DMI Fungicides

**Figure 4:**
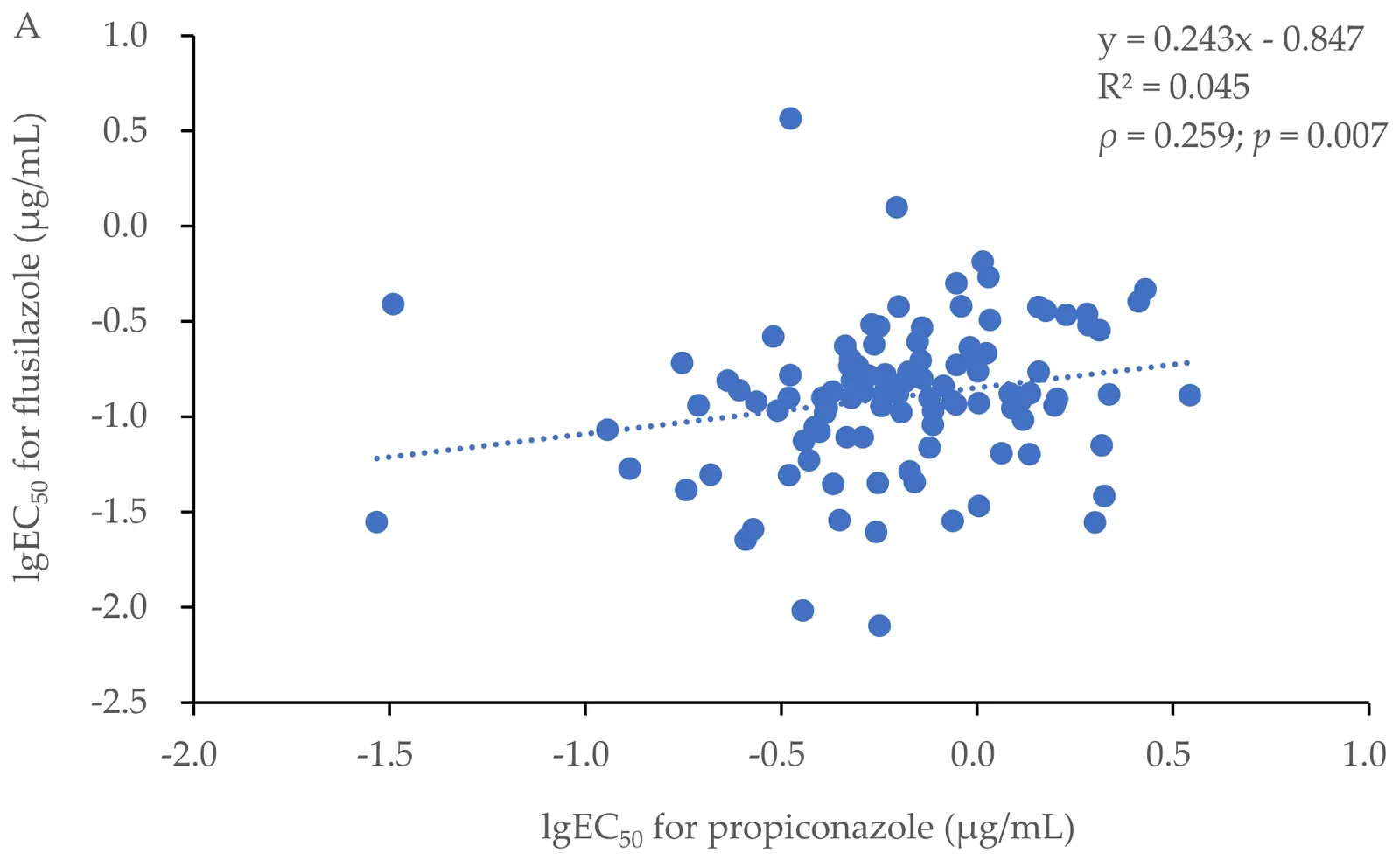

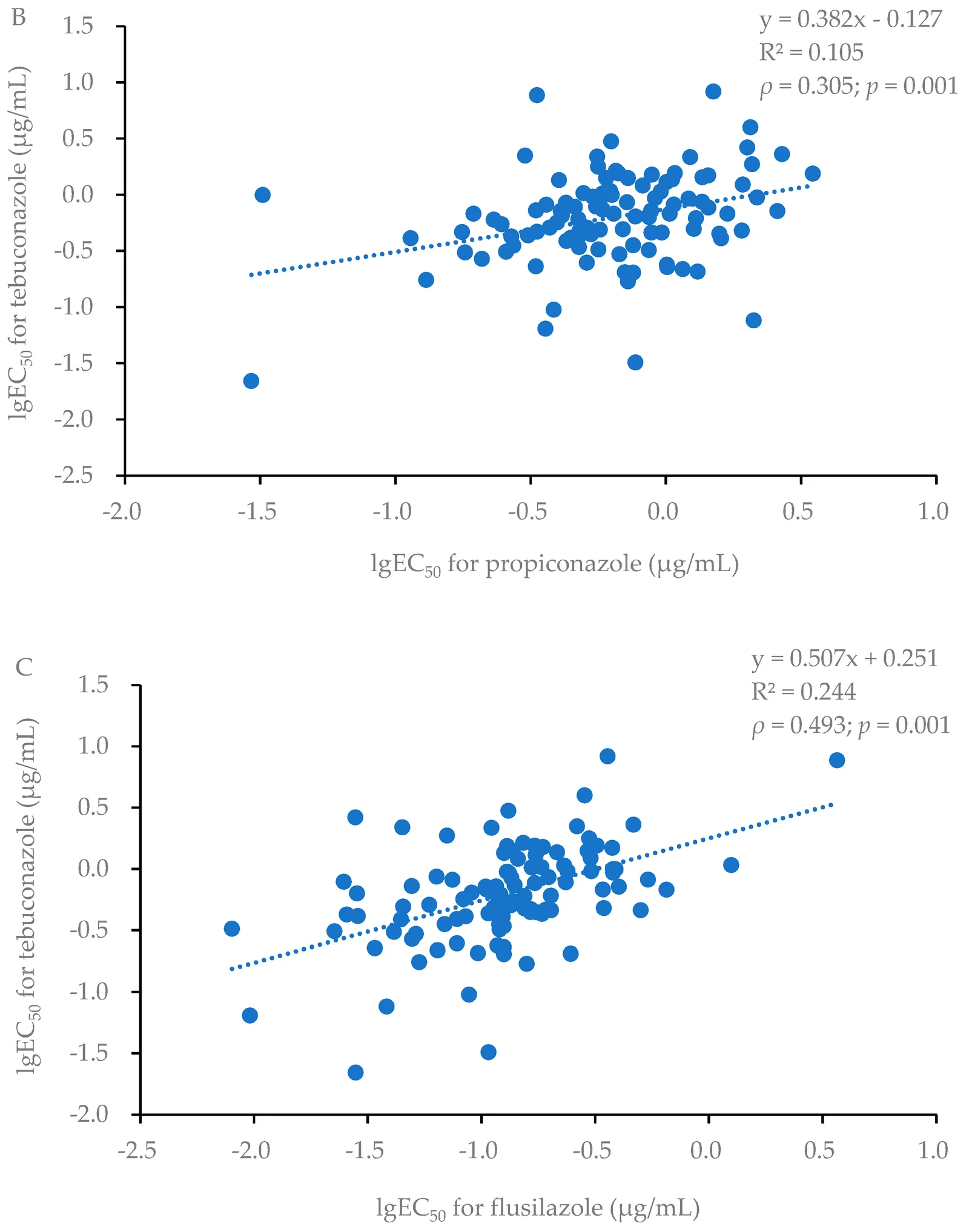
Correlation of sensitivity to propiconazole, flusilazole and tebuconazole based on lgEC_50_ values of *B. oryzae* isolates. (**A**) Correlation in sensitivity of propiconazole with flusilazole based on lgEC_50_ values of 108 *B. oryzae* isolates. (**B**) Correlation in sensitivity of propiconazole with tebuconazole based on lgEC_50_ values of 108 *B. oryzaes* isolates. (**C**) Correlation in sensitivity of flusilazole with tebuconazole based on lgEC_50_ values of 108 *B. oryzae* isolates. *ρ*, Spearman’s rho.

There was a statistically significant and weakly to moderately positive correlation between propiconazole and flusilazole (*n* = 108, *ρ* = 0.259, *p* = 0.007) (Figure 4A), propiconazole and tebuconazole (*n* = 108, *ρ* = 0.305, *p* = 0.001) (Figure 4B), and flusilazole and tebuconazole (*n* = 108, *ρ* = 0.493, *p* = 0.001) (Figure 4C), as determined from lgEC_50_ values, suggesting cross-resistance among the three DMI fungicides.

### 3.4. Resistance Level and Stability of Field-Resistant Isolates of B. oryzae

Seven field-resistant isolates of *B. oryzae* were obtained based on the RF analysis (Table 2), including one isolate with low resistance to propiconazole (JJBO5), one isolate with low resistance to flusilazole (FSBO4), one isolate with moderate resistance to flusilazole (FSBO10), one isolate with moderate resistance to both flusilazole and tebuconazole (JJBO15), one isolate with moderate resistance to tebuconazole (LCBO2), and two isolates with low resistance to tebuconazole (FSBO59 and DFBO1) (Table 2). Resistance frequencies of 108 *B. oryzae* isolates to propiconazole, flusilazole and tebuconazole were 0.93%, 2.78% and 3.70%, respectively.

After eight transfers on fungicide-free PDA media, the RF values of each resistant isolate from the field showed significant decline, among which six resistant isolates returned to the level of sensitivity and one resistant isolate slipped from a moderate to low level, and the decline in resistance was 39.67% for propiconazole, 49.88–81.22% for flusilazole and 35.26–87.98% for tebuconazole, suggesting that the resistance of *B. oryzae* to the three DMI fungicides could not be stably inherited (Table 3).

### 3.5. Fitness of Field-Resistant Isolates of B. oryzae

For the fitness evaluation, seven field-resistant isolates of *B. oryzae* were evaluated based on the parameters of mycelial growth, mycelial dry weight and pathogenicity. For mycelial growth, all the flusilazole- and tebuconazole-resistant isolates were remarkably slower (*p* < 0.05) than the sensitive isolates except for propiconazole-resistant isolate JJBO5 (Table 4). Mycelium growth was faster for propiconazole-resistant isolate JJBO5 compared with all the flusilazole- and tebuconazole-resistant isolates and the sensitive isolate JJBO9. Compared to the two sensitive isolates under the same conditions, all of the seven resistant isolates had a significant decline (*p* < 0.05) in mycelial dry weight and lesion size (Table 4).

### 3.6. Protective and Curative Activities of Three DMI Fungicides

It was shown in the artificial inoculation potted plant assay that 250 g/L of propiconazole EC, 400 g/L of flusilazole EC and 25% tebuconazole WP at the highest recommended doses (500 μg/mL, 66.67 μg/mL and 25 μg/mL, respectively) achieved an excellent protective control efficacy of 100% against *B. oryzae* with application 24 h before inoculation (Table 5). There was no significant difference (*p* > 0.05) in the protective efficacy among these three fungicides to the sensitive and fungicide-resistant isolates.

For the curative activity, propiconazole, flusilazole and tebuconazole showed poor control efficacies of less than 40% when applied 24 h after inoculation, which were significantly lower than the protective control efficacies (Table 5). Curative control efficacies of fungicide-sensitive isolates were a little higher than those of fungicide-resistant isolates, but except for tebuconazole-resistant isolate FSBO59, the difference was not statistically significant (*p* > 0.05).

## 4. Discussion

Bananas are threatened by several *Bipolars* species, causing eyespot that negatively affects yield and quality, and pathogenic species vary by distinct region [2,3,4,5,10]. Among them, the pathogen *B. oryzae* was reported to cause severe eyespot on the banana cultivar ‘Huangdi banana’ in Hainan Island, China [2,8]. DMI fungicides such as propiconazole, flusilazole and tebuconazole have been used for foliage diseases of banana in China since 2006, 2001 and 2010, respectively (China Pesticide Information Network, http://www.chinapesticide.org.cn (accessed on 16 January 2024)). Despite extensive use of these fungicides, the prevalence of eyespot disease of ‘Huangdi banana’ is increasing across Hainan Island, and limited information exists on the fungicide sensitivity of *B. oryzae* in this region. DMI fungicides were categorized as medium risk for resistance development by the Fungicide Resistance Action Committee [23]. Establishing a sensitivity baseline is essential for early detection of sensitivity shifts, resistance risk assessment and resistance monitoring, and will be helpful for the design of effective disease management strategies.

It is not easy to sporulate *B. oryzae* under artificial culture conditions [8,24,25], which hinders the testing of the sensitivity of the pathogen to fungicides. Therefore, in this study, a total of 108 isolates of *B. oryzae* were tested for their sensitivity to propiconazole, flusilazole and tebuconazole with the method of mycelial growth inhibition on PDA. Results showed that the tested isolates of *B. oryzae* showed a relatively broad range of EC_50_ values for propiconazole, flusilazole and tebuconazole, with a VF range (MaxEC_50/_MinEC_50_) of 119.27–458.10, which is similar to the broad distributions reported for *Cochliobolus heterostrophus* (anamorph: *Bipolaris maydis*) to propiconazole [26] and tebuconazole [27,28], unlike the narrower distributions reported for *B. maydis* to flusilazole [29].

Some studies have indicated that geographic locations have an effect on the sensitivity of *C. heterostrophus* [26] and *Monilia yunnanensis* [30] to DMI fungicides, and others have indicated that geographic locations have no effect on the sensitivity of *Sclerotinia sclerotiorum* [31] and *Fusarium graminearum* [32] to DMI fungicides. Our results indicated that there was no statistically significant difference (*p* > 0.05) in sensitivity to these three DMI fungicides among *B. oryzae* populations collected from different locations (Table 1); thus, propiconazole, flusilazole and tebuconazole sensitivity data were merged for isolates from different locations to determine a single baseline sensitivity. Analysis of sensitivity distribution signaled a reduced sensitivity or a potential resistance to the three DMI fungicides in isolates from ‘Huangdi banana’. However, most *B. oryzae* isolates tested were still sensitive to propiconazole, flusilazole and tebuconazole (82.41%, 82.41% and 80.56%, respectively). It is ideal that pathogen isolates with no prior exposure to the targeted fungicide(s) are used to establish baseline sensitivities. Although the sensitivity data in this study cannot be considered the natural baseline sensitivity to propiconazole, flusilazole and tebuconazole, as the isolates of *B. oryzae* tested had been exposed previously to DMI fungicides, our data still provide useful future reference points for resistance monitoring campaigns for these three DMI fungicides.

To the best of our knowledge, there have been no reports on propiconazole-, flusilazole- or tebuconazole-resistant isolates of *B. oryzae*. In this study, based on 108 *B. oryzae* isolates from banana, we first detected propiconazole, flusilazole and tebuconazole, field-resistant isolates from Hainan Island, China, with an RF range of 5.20–31.14, belonging to the low to medium resistance level. No highly resistant isolates of *B. oryzae* to the three DMI fungicides were detected. Previous studies demonstrated that there were fitness penalties in DMI-resistant isolates in *Colletotrichum truncatum* [33] and *Cercospora beticola* [34]. Similarly, in this study, a fitness penalty was observed in all seven field-resistant isolates of *B. oryzae*, which showed significantly reduced fitness in terms of mycelial growth rates, mycelial dry weight and pathogenicity in comparison to the sensitive isolates, with the exception of the mycelial growth of isolate JJBO5 (Pro^LR^Flu^S^Teb^S^). Based on the results above in this study, application of the three DMI fungicides to control banana eyespot disease is presently at low risk, and their usage is still a good choice in regions where *B. oryzae* is the predominant species causing banana eyespot. However, the development of resistance in *B. oryzae* to propiconazole, flusilazole and tebuconazole should be extensively monitored because these fungicides have been widely used. Furthermore, the sensitivity data from this study will be useful in determining whether resistance is developing.

It is generally wise to accept that cross-resistance is present between DMI fungicides active against the same fungus [23]. In fact, cross-resistance among DMI fungicides has been reported in various fungi, such as *C. heterostrophus* [26], *Fusarium culmorum* [35], and *S. sclerotiorum* [36]. Pairwise comparisons in this study indicated significant and positive associations among the three DMI fungicides tested, providing evidence that cross-resistance relationships exist in *B. oryzae* to propiconazole, flusilazole and tebuconazole. Therefore, the sensitivity of *B. oryzae* to propiconazole, flusilazole, tebuconazole and other DMI fungicides should be closely monitored in the future. In addition, it is necessary at present to assess the efficacy of these three DMI fungicides against any DMI-resistant isolates of *B. oryzae* collected in the field in Hainan Island.

DMI fungicides have been reported to be efficacious systematic azole fungicides, with both protective and curative activity against a wide range of higher phytopathogenic fungi [18,37]. In addition, precise timing of the application of fungicide is also critically important for disease control. Some studies have found the protective efficacy of DMI fungicides was greater than their curative efficacy [26,31,38]. Our results are consistent with the observations that protective application of DMI fungicides is more efficacious for controlling banana eyespot compared to a curative application. These findings indicate that propiconazole, flusilazole and tebuconazole should be used before the occurrence of banana eyespot.

In conclusion, this study first evaluated the sensitivity status of *B. oryzae* in Hainan Island, China, to propiconazole, flusilazole and tebuconazole, and established the sensitivity baselines, which could serve as an important basis for monitoring the development of resistance of *B. oryzae* to these three DMI fungicides in the field. Seven field-resistant isolates of *B. oryzae* to the three DMI fungicides were detected, but they all suffered fitness penalties in comparison with the sensitive isolate. We speculate that *B. oryzae* exhibited a low level of resistance risk to the three DMI fungicides. Propiconazole, flusilazole and tebuconazole exhibited good protective efficacy against banana eyespot in potted experiments; however, cross-resistance existed among them. Therefore, in order to retard the development of resistance, integrated pathogen management strategies such as the use of resistant varieties, biological control agents, crop rotations, and mixtures or rotations of fungicides with different modes of action should be applied to ensure sustainable management of *B. oryzae*.

## Figures and Tables

**Table 1:** Sensitivity of *B. oryzae* isolates to propiconazole, flusilazole and tebuconazole from different locations of Hainan Island, China.

Origin	No. of Isolates	EC_50_ (μg/mL) ^A^							
		Propiconazole			Flusilazole			Tebuconazole	
		Range	Mean ^B^		Range	Mean ^B^		Range	Mean ^B^
Chengmai	72	0.029–3.495	0.766 ± 0.074 a		0.008–3.665	0.208 ± 0.053 a		0.022–7.695	0.906 ± 0.125 a
Lingao	24	0.302–2.585	1.061 ± 0.126 a		0.010–0.540	0.210 ± 0.030 a		0.064–8.288	1.252 ± 0.333 a
Danzhou	11	0.130–1.601	0.745 ± 0.134 a		0.053–0.380	0.182 ± 0.034 a		0.175–1.631	0.642 ± 0.130 a
Ding’an	1	0.372	0.372 ± 0.000		0.059	0.059 ± 0.000		0.510	0.510± 0.000
Total	108	0.029–3.495	0.826 ± 0.059		0.008–3.665	0.204 ± 0.036		0.022–8.288	0.952 ± 0.113
*VF* ^C^		119.27			458.10			376.73	
F (*p*) value ^D^		1.67 (0.178)			0.07 (0.978)			0.86 (0.463)	

^A^ EC_50_, the effective concentration of the fungicide causing a 50% inhibition of mycelial growth as compared with the control. ^B^ Data are presented as the mean ± standard error of EC_50_ from different locations. Same lowercase letter (a) indicates no significant difference (*p* > 0.05) according to ANOVA. ^C^ VF, variation factor (maximum EC_50_)/(minimum EC_50_). ^D^ *F* and *p* values indicate the results of an ANOVA comparing the EC_50_ for each DMI fungicide between the locations within Hainan Island at the *p* = 0.05 level.

**Table 2:** Resistance level of propiconazole, flusilazole and tebuconazole field-resistant isolates of *B. oryzae*.

Isolate	Origin	Propiconazole				Fusilazole				Tebuconazole		
		EC_50_ (μg/mL) ^A^	RF ^B^	Resistance Level ^C^		EC_50_ (μg/mL) ^A^	RF ^B^	Resistance Level ^C^		EC_50_ (μg/mL) ^A^	RF ^B^	Resistance Level ^C^
JJBO5	Chengmai	3.495	5.88	LR		0.130	1.10	S		1.539	2.68	S
FSBO4	Chengmai	1.034	1.74	S		0.651	5.53	LR		0.677	1.18	S
FSBO10	Chengmai	0.623	1.05	S		1.255	10.66	MR		1.077	1.88	S
JJBO15	Chengmai	0.334	0.56	S		3.665	31.14	MR		7.695	13.42	MR
LCBO2	Linggao	1.499	2.52	S		0.359	3.05	S		8.288	14.45	MR
FSBO59	Chengmai	2.055	3.46	S		0.284	2.41	S		3.977	6.94	LR
DFBO1	Chengmai	0.628	1.06	S		0.131	1.12	S		2.982	5.20	LR

^A^ EC_50_, the effective concentration of the fungicide causing a 50% inhibition of mycelial growth as compared with the control. ^B^ RF, resistance factor, the ratio of the EC_50_ values of resistant isolates relative to the mean EC_50_ values of sensitive isolates. The mean EC_50_ values of 89 propiconazole-sensitivity, 89 flusilazole-sensitivity and 87 tebuconazole-sensitivity isolates were 0.594 μg/mL, 0.118 μg/mL and 0.574 μg/mL, respectively. ^C^ S, LR and MR indicate sensitive, low resistance and moderate resistance, respectively.

**Table 3:** Stability of propiconazole, flusilazole, and tebuconazole field-resistant isolates of *B. oryzae*.

Isolate	EC_50_ (μg/mL) ^A^									RF ^B^							
	Propiconazole			Fusilazole			Tebuconazole			Propiconazole			Fusilazole			Tebuconazole	
	F0	F8		F0	F8		F0	F8		F0	F8		F0	F8		F0	F8
JJBO5	3.495	2.112		0.130			1.539			5.88	3.55		1.10	–		2.68	–
FSBO4	1.034			0.651	0.326		0.677			1.74	–		5.53	2.77		1.18	–
FSBO10	0.623			1.255	0.574		1.077			1.05	–		10.66	4.88		1.88	–
JJBO15	0.334			3.665	0.686		7.695	3.868		0.56	–		31.14	5.83		13.42	6.74
LCBO2	1.499			0.359			8.288	0.996		2.52	–		3.05	–		14.45	1.74
FSBO59	2.055			0.284			3.977	2.575		3.46	–		2.41	–		6.94	4.49
DFBO1	0.628			0.131			2.982	0.906		1.06	–		1.12	–		5.20	1.58

^A^ 50% effective concentration (EC_50_) of F0 (the zeroth generation) and F8 (the eighth generation). ^B^ RF, resistance factor, the ratio of the EC_50_ values of resistant isolates relative to the mean EC_50_ values of sensitive isolates. The mean EC_50_ values of 89 propiconazole-sensitivity, 89 flusilazole-sensitivity and 87 tebuconazole-sensitivity isolates were 0.594 μg/mL, 0.118 μg/mL and 0.574 μg/mL, respectively.

**Table 4:** Fitness of propiconazole, flusilazole and tebuconazole field-resistant isolates of *B. oryzae*.

Isolate ^A^	Phenotype ^B^	Mean Mycelial Growth Rate (cm/d) ^C^	Mycelial Dry Weight (g) ^C^	Mean Lesion Diameter (cm) ^C^
JJBO5	Pro^LR^Flu^S^Teb^S^	1.96 ± 0.050 a	0.061 ± 0.029 c	1.21 ± 0.026 d
FSBO4	Pro^S^Flu^LR^Teb^S^	1.11 ± 0.025 c	0.094 ± 0.036 b	1.73 ± 0.037 b
FSBO10	Pro^S^Flu^MR^Teb^S^	1.14 ± 0.035 c	0.074 ± 0.014 c	1.09 ±0.024 d
JJBO15	Pro^S^Flu^MR^Teb^MR^	0.84 ± 0.032 d	0.039 ± 0.011 d	1.23 ± 0.026 d
LCBO2	Pro^S^Flu^S^Teb^MR^	1.16 ± 0.014 c	0.070 ± 0.017 c	1.43 ± 0.039 c
FSBO59	Pro^S^Flu^S^Teb^LR^	1.17 ± 0.025 c	0.047 ± 0.014 d	1.19 ± 0.021 d
DFBO1	Pro^S^Flu^S^Teb^LR^	1.29 ± 0.011 c	0.052 ± 0.013 d	1.14 ± 0.017 d
JJBO8	Pro^S^Flu^S^Teb^S^	1.99 ± 0.032 a	0.131 ± 0.024 a	2.46 ± 0.044 a
JJBO9	Pro^S^Flu^S^Teb^S^	1.56 ± 0.016 b	0.145 ± 0.023 a	2.48 ± 0.034 a

^A^ JJBO8 and JJBO9, sensitive to the three fungicides, served as controls. The EC_50_ values of JJBO8 to propiconazole, flusilazole and tebuconazole were 0.673 μg/mL, 0.052 μg/mL and 0.296 μg/mL, with resistance factors (RFs) of 1.133, 0.441, and 0.516, respectively. The EC_50_ values of JJBO9 to propiconazole, flusilazole and tebuconazole were 0.772 μg/mL, 0.107 μg/mL and 0.032 μg/mL, with RFs of 1.300, 0.907, and 0.516, respectively. ^B^ Pro, propiconazole; Teb, tebuconazole; Flu, flusilazole. Superscripts S, LR and MR indicate sensitive, low resistance and moderate resistance, respectively. ^C^ Data are presented as the mean ± standard error (*n* = 3) of two independent experiments. Different lowercase letters (a, b, c, d) indicate significantly differences (*p* < 0.05) according to ANOVA.

**Table 5:** Protective and curative activity of propiconazole, flusilazole and tebuconazole against banana eyespot on greenhouse potted banana plants.

Isolate ^A^	Treatment			Protective Activity ^C^			Curative Activity ^C^	
	Fungicide ^B^	Active Ingredient (μg/mL)		Mean Lesion Diameter (cm)	Control Efficacy (%)		Mean Lesion Diameter (cm)	Control Efficacy (%)
JJBO5	250 g/L Pro EC	500		0 ± 0 c	100.0 ± 0 a		1.05 ± 0.21 b	25.00 ± 6.1 a
	CK	–		1.51 ± 0.15 b	–		1.40 ± 0.12 b	–
JJBO8	250 g/L Pro EC	500		0 ± 0 c	100.0 ± 0 a		1.23 ± 0.38 b	32.79 ± 7.1 a
	CK	–		2.61 ± 0.14 a	–		1.83 ± 0.34 a	–
JJBO9	250 g/L Pro EC	500		0 ± 0 c	100.0 ± 0 a		1.38 ± 0.27 b	28.50 ± 2.7 a
	CK	–		2.64 ± 0.12 a	–		1.93 ± 0.23 a	–
FSBO4	400 g/L Flu EC	66.67		0 ± 0 d	100.0 ± 0 a		1.04 ± 0.19 d	34.18 ± 9.4 a
	CK	–		2.06 ± 0.21 b	–		1.58 ± 0.20 b	–
FSBO10	400 g/L Flu EC	66.67		0 ± 0 d	100.0 ± 0 a		0.85 ± 0.13 d	34.11 ± 6.1 a
	CK	–		1.45 ± 0.18 c	–		1.29 ± 0.20 c	–
JJBO15	400 g/L Flu EC	66.67		0 ± 0 d	100.0± 0 a		0.98 ± 0.15 d	30.00± 5.9 a
	CK	–		1.59 ± 0.14 c	–		1.40 ± 0.19 c	–
JJBO8	400 g/L Flu EC	66.67		0 ± 0 d	100.0 ± 0 a		1.18 ± 0.21 c	35.52 ± 9.1 a
	CK	–		2.61 ± 0.14 a	–		1.83 ± 0.34 a	–
JJBO9	400 g/L Flu EC	66.67		0 ± 0 d	100.0 ± 0 a		1.29 ± 0.12 c	33.16 ± 6.0 a
	CK	–		2.64 ± 0.12 a	–		1.93 ± 0.34 a	–
JJBO15	25% Teb WP	25		0 ± 0 d	100.0± 0 a		0.94 ± 0.18 d	32.86 ± 8.5 a
	CK	–		1.55 ± 0.17 c	–		1.40 ± 0.19 c	–
LCBO2	25% Teb WP	25		0 ± 0 d	100.0± 0 a		0.95 ± 0.16 d	27.48 ± 3.5 ab
	CK	–		1.76 ± 0.17 bc	–		1.31 ± 0.13 c	–
FSBO59	25% Teb WP	25		0 ± 0 d	100.0± 0 a		1.05 ± 0.23 d	22.79 ± 7.4 b
	CK	–		1.53 ± 0.07 c	–		1.36 ± 0.07 c	–
DFBO1	25% Teb WP	25		0 ± 0 d	100.0± 0 a		1.01 ± 0.12 d	27.34 ± 2.5 ab
	CK	–		1.54 ± 0.05 c	–		1.39 ± 0.14 c	–
JJBO8	25% Teb WP	25		0 ± 0 d	100.0 ± 0 a		1.20 ± 0.21 c	34.43 ± 4.4 a
	CK	–		2.61 ± 0.14 a	–		1.83 ± 0.34 a	–
JJBO9	25% Teb WP	25		0 ± 0 d	100.0± 0 a		1.30 ± 0.11 c	32.64 ± 3.6 a
	CK	–		2.64 ± 0.12 a	–		1.93 ± 0.23 a	–

^A^ JJBO8 and JJBO9, sensitive to the three fungicides, served as controls. ^B^ Pro, propiconazole; Flu, flusilazole; Teb, tebuconazole. EC, emulsifiable concentrate; WP, wettable powder; CK, control. ^C^ Data are presented as the mean ± standard error (*n* = 3) of two independent experiments. Different lowercase letters (a, b, c, d) indicate significant differences (*p* < 0.05) according to ANOVA.

## Data Availability

The data presented in this study are available within the article.
